# Frequency of circulating CD8+CD73+T cells is associated with survival in nivolumab-treated melanoma patients

**DOI:** 10.1186/s12967-020-02285-0

**Published:** 2020-03-11

**Authors:** Mariaelena Capone, Federica Fratangelo, Diana Giannarelli, Claudia Sorrentino, Roberta Turiello, Serena Zanotta, Domenico Galati, Gabriele Madonna, Marilena Tuffanelli, Luigi Scarpato, Antonio M. Grimaldi, Assunta Esposito, Rosa Azzaro, Antonio Pinto, Ernesta Cavalcanti, Aldo Pinto, Silvana Morello, Paolo A. Ascierto

**Affiliations:** 1 Istituto Nazionale Tumori – IRCCS - Fondazione “G. Pascale”, Naples, Italy; 2 Istituto Nazionale Tumori Regina Elena, IRCCS, Rome, Italy; 3 Department of Pharmacy, University of Salerno, Via Giovanni Paolo II, 132, 84084 Fisciano, SA Italy; 4 PhD Program in Drug Discovery and Development, Department of Pharmacy, University of Salerno, Fisciano, Italy; 5 Present Address: Department of Medicine and Surgery, University of Parma, Parma, Italy

**Keywords:** Immunotherapy, Nivolumab, Metastatic melanoma, CD73, Circulating CD8+ lymphocytes

## Abstract

**Background:**

PD-1 blocking agents, such as nivolumab, have demonstrated clear anti-tumor effects and clinical benefits in a subset of patients with advanced malignancies. Nonetheless, more efforts are needed to identify reliable biomarkers for outcome, to correctly select patients who will benefit from anti-PD-1 treatment. The aim of this study was to investigate the role of peripheral CD8+T cells expressing CD73, involved in the generation of the immune suppressive molecule adenosine, in predicting outcome after nivolumab treatment in advanced melanoma patients.

**Methods:**

PBMCs from 100 melanoma patients treated with nivolumab were collected at National Cancer Institute “G. Pascale” of Naples. Frequencies of CD8+ lymphocytes phenotypes were assessed by flow cytometry at baseline before nivolumab treatment, along with clinical characteristics and blood count parameters. Healthy controls (n = 20) were also analysed. Percentages of baseline T cells expressing PD-1 and CD73 were correlated with outcome after nivolumab treatment.

**Results:**

Melanoma patients presented a lower frequency of total circulating CD8+ lymphocytes than control subjects (p = 0.008). Patients with low baseline percentage of circulating CD8+PD-1+CD73+ lymphocytes (< 2.3%) had better survival (22.4 months *vs* 6.9 months, p = 0.001). Patients (39%) with clinical benefit from nivolumab therapy presented a significantly lower frequency of circulating CD8+PD-1+CD73+ lymphocytes than patients who progressed to nivolumab treatment (p = 0.02).

**Conclusions:**

Our observations suggest that baseline CD73 expression on circulating CD8+PD-1+ lymphocytes appear a promising biomarker of response to anti-PD-1 treatment in melanoma patients. Further investigations are needed for validation and for clarifying its role as prognostic or predictive marker.

## Background

T cells, especially CD8+T cells, are critical players in cancer control, being stimulated by tumor-associated antigens presented by Class I MHC molecules. In the tumor microenvironment the activity of CD8+T cells can be hampered by immunosuppressive molecules, such as programmed cell death protein-1 (PD-1), cytotoxic T lymphocyte antigen-4 (CTLA-4), T cell immunoglobulin and mucin domain-containing protein 3 (TIM3), lymphocyte activation gene 3 protein (LAG3) and T-cell immune-receptor with immunoglobulin and ITIM domains (TIGIT), exhibiting an exhausted phenotype and function [1].

PD-1 is one of the major inhibitory receptor expressed on activated T cells and other cell types. Binding of PD-1 to its ligands PD-L1 or PD-L2 suppresses T cell receptor (TCR)/CD28 signalling pathway resulting in inhibition of T-cell proliferation and cytokine secretion [2]. The anti-PD-1 monoclonal antibodies (mAbs) have been approved in monotherapy and combination therapy for treatment of metastatic melanoma, proving impressive clinical benefit [3–6] and long-term survival for up to 5 years [7, 8]. However, a high number of patients still fail to have a clinical response from anti-PD-1 therapy, and in addition patients experienced, sometimes, serious treatment-related side effects. Therefore, more efforts are needed to identify reliable biomarkers for outcome, to correctly select patients who will benefit from anti-PD-1 mAb treatment and possibly useful to develop strategies to enhance the efficacy of checkpoint inhibitors.

Extracellular adenosine is emerging as a highly clinically relevant mediator of tumor immune escape [9–13]. Extracellular adenosine derives from adenosine triphosphate (ATP), being de-phosphorylated first into adenosine-5′-diphosphate (ADP) and then into adenosine monophosphate (AMP) through the membrane-bound extracellular ecto-nucleotidase CD39 [14, 15]. Alternatively, AMP can be generated from ATP by the ecto-phosphodiesterase/pyrophosphatase CD203a or from ADP-ribose (ADPR) (generated by nicotinamide adenine dinucleotide, NAD+) by the NAD+ glycohydrolase CD38 [16]. AMP from both cascades is then de-phosphorylated into adenosine and inorganic phosphate through the 5′-nucleotidase CD73 [14, 15]. CD73 is expressed by cancer cells, endothelial cells, exosomes, and immune cell populations: predominantly T cells and, to a lesser extent, B cells, natural killer (NK) and myeloid-derived suppressor cells (MDSCs) [12]. Within the tumor microenvironment, under hypoxic conditions, CD73-derived adenosine accumulates and potently suppresses T cell-mediated anti-tumor immune responses, mainly through stimulation of the cyclic AMP-elevating adenosine receptor A2A, reducing cytokines production, proliferation and cytotoxicity [17–22]. CD73 is highly expressed in several types of human cancer and often its expression is associated with poor prognosis in different cancer types [23–27]. Elevated activity of soluble CD73 in the peripheral blood of melanoma patients is also associated with low response rate to nivolumab and shorter survival [28]. Pharmacological blockade of CD73 promotes anti-tumor immune responses and inhibits tumor metastasis, by reducing the accumulation of adenosine [13], and enhances the efficacy of anti-tumor agents, including anti-PD-1 or anti-CTLA-4 mAbs [29, 30]. At present, new agents that reduce the generation of extracellular adenosine or its activity are undergoing first-in-human clinical trials in patients with malignancies, alone or in combination with immunotherapeutic agents [reviewed in Ref 10, 13].

Given the crucial role of T cells as harms of cancer immunotherapeutic agents, whose activity is influenced by co-expression of immune suppressive mediators and/or receptors, in this study we analysed the frequency of circulating CD8+ lymphocytes expressing CD73, PD-1 or both in metastatic melanoma patients treated with nivolumab, in comparison with healthy subjects. Furthermore, we evaluated the role of circulating CD8+T cells expressing CD73 in clinical response to nivolumab and overall survival (OS).

## Materials and methods

### Patients and human samples

Peripheral blood from stage III or IV melanoma patients treated with nivolumab was collected at the Unit of Melanoma, Cancer Immunotherapy and Innovative Therapies, of Istituto Nazionale Tumori IRCCS Fondazione “G. Pascale”, Naples, Italy after obtaining the approval of Ethics Committee and a signed informed consent from patients. In addition, peripheral blood from healthy subjects was collected at the Transfusion and Stem Cell Transplantation Unit, of the same Institute. Peripheral blood mononuclear cells (PBMCs) were isolated from blood of melanoma patients and healthy subjects by Ficoll density gradient following standardized protocols and then cryo-preserved at − 80 °C until use.

Blood samples from melanoma patients were collected before starting nivolumab therapy (baseline), administered at the dosage of 3 mg/kg every 2 weeks until disease progression or unacceptable toxicity appeared. For all patients, clinical data, including serum lactate dehydrogenase (LDH), complete blood count, BRAF status, brain metastasis, lines of prior treatment were collected before starting nivolumab treatment and until last follow-up. Tumor assessment was performed at baseline, at week 12, and every 12 weeks thereafter, and clinical response was classified according to response evaluation criteria in solid tumors (RECIST) as complete response (CR), partial response (PR), stable disease (SD) or progressive disease (PD) [31].

### Flow cytometry analysis

Frozen PBMCs were thawed, briefly rested and then incubated with antibodies. Subpopulations of PBMCs were analysed using the following antibodies: CD3-V500, CD8-APC Clone BW135/80, PD-1-PE Clone PD1.3.1.3 (all from MiltenyiBiotecS.r.l.) and CD73 PE-Cy7 Clone AD2 (BioLegend UK Ltd). Samples Data were acquired using a FACSAria II (Becton–Dickinson, USA). Cell viability was assessed by 7-AAD staining. Dead cells were excluded by selecting only 7-AAD-negative cells. The population of lymphocytes was identified using a morphological gating on forward/side light scatters (FSC-A and SSC-A, respectively) and further gated by the expression of CD3 and CD8. The expression of CD73 and/or PD-1 were determined on the population of interest CD3+CD8+T cells (Additional file 1: Fig. S1). Data were analysed using Kaluza 1.2 software (Beckman Coulter).

### Statistical analysis

Patients baseline characteristics were described using descriptive statistics. Differences in cells subsets frequencies between patients and controls were evaluated with the Mann–Whitney U test. The Kaplan–Meier method was used to estimate the disease-associated survival probabilities of patients and differences evaluated by the log-rank tests at cut-off values identified with the median. OS time was calculated from the date of the first dose of nivolumab to the date of death (due to melanoma) or censored at the date of the last follow-up. Cox regression analysis for survival was performed and reported as hazard ratio (HR). Analyses were performed with GraphPad Prism 7.0 or IBM SPSS version 21.0. p values < 0.05 were considered statistically significant.

## Results

### Patients characteristics

A total of 100 melanoma patients treated with nivolumab were included. Baseline characteristics of patients are shown in Table 1. The median age was 62 years; 53 patients were male and 47 were female. According to the 7th edition of American Joint Committee on Cancer (AJCC) [32] 8 patients were assigned to category M1a, 12 in M1b, 78 in M1c, and M0 for 2 patients. *BRAF* mutational status was known for 97 patients: 43 patients (43%) had *BRAF*-mutated melanoma, 54 patients (54%) were wild-type for *BRAF*. Brain metastasis were present in 28/100 patients (28%). Twenty-seven patients received nivolumab as first-line therapy; whereas 73 patients had previously received ipilimumab alone or in combination with a BRAF inhibitor. Of 100 patients, 58 patients (58%) had PD, 22 patients (22%) had SD, 20 patients (20%) had PR or CR. In order to define which subsets of cells population were associated with clinical response, patients with CR, PR or SD for greater than 6 months (39/100 patients) were arbitrarily grouped into patients with clinical benefit (CB) while no-clinical benefit group (NCB) exhibit PD or SD for lesser than 6 months (61/100 patients). The mean duration of SD for patients with clinal benefit prior progression was 16.4 months. The median follow-up for this study was 11 months.

**Table 1:** Patients characteristics

Variable	Patients (n = 100)
Gender, *n* (%), female/male	47 (47)/53 (53)
Age, years, median (range)	62 (28–90)
LDH, *n* (%)	
Normal	60 (60)
Elevated	34 (34)
Unknown	6 (6)
*B*-*RAF*, *n* (%)	
Mutation	43 (43)
Wild-type	54 (54)
Unknown	3 (3)
Brain metastasis, *n* (%)	28 (28)
M category (AJCC), *n (%)*	
M0	2 (2)
M1a	8 (8)
M1b	12 (12)
M1c	78 (78)
Line of treatment, *n* (%)	
First line	27 (27)
> 2	73 (73)

A total of 20 healthy subjects (male, n = 15; female, n = 5), aged > 18 years, were also analysed.

### Frequencies of peripheral CD8+CD73+T cells in melanoma patients and healthy subjects

**Fig. 1:**
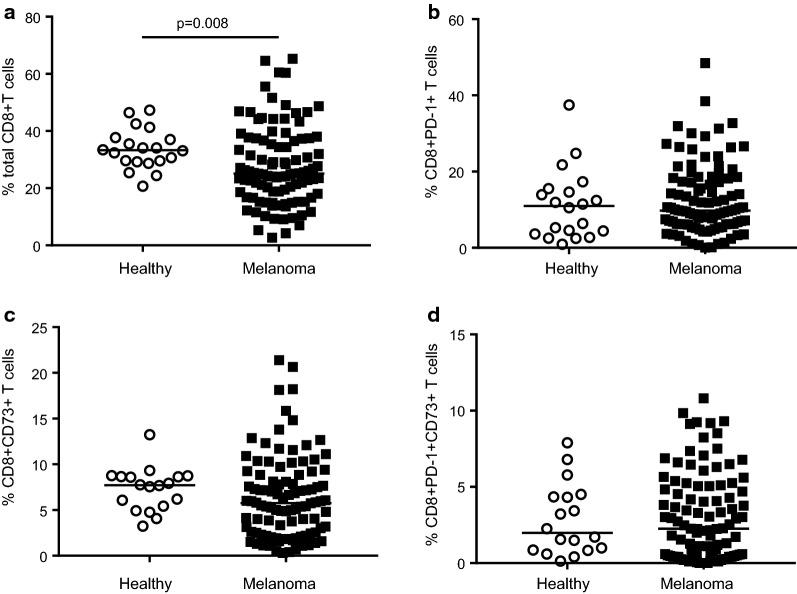
CD3+CD8+T cells in PBMCs. **a** Percentage of total CD8+ cells, **b** percentage of CD8+PD-1+ cells, **c** percentage of CD8+CD73+ cells and **d** percentage of CD8+PD-1+CD73+ cells in melanoma patients versus healthy subjects (controls n = 20, melanoma n = 100). Statistical analysis was performed with Mann–Whitney test. Line indicates the median

We investigated the frequency of peripheral CD8+ lymphocytes subsets positive to CD73 and/or PD-1 by flow cytometry analysis. The characterization of these cells was performed in samples from blood of melanoma patients before starting nivolumab therapy, as stated above, and in healthy subjects. Patients with melanoma presented a decreased frequency of total CD8+T cells compared with control subjects (25.1% *vs* 33.28%, respectively; p = 0.008) (Fig. 1a). CD8+PD-1+ lymphocytes were present at a median frequency of 9.8% in melanoma patients and 11.0% in healthy subjects (Fig. 1b). The frequency of CD8+ lymphocytes subset expressing CD73 was 5.8% median and 7.7% median in melanoma patients and healthy donors, respectively (Fig. 1c). CD73+ cells among CD8+PD-1+ cells were 2.3% in melanoma patients and 1.97% for healthy donors (Fig. 1d).

### Association of pre-treatment CD8+CD73+T cells frequency with overall survival in patients treated with nivolumab

Frequency of peripheral blood CD8+ cells in melanoma patients together with available blood counts and clinical characteristics were analysed to identify pre-treatment factors associated with OS by Cox regression analysis. We found significant negative correlations with OS for very high LDH levels (ratio LDH value/LDH normal > 2) (p < 0.0001), brain metastasis (p < 0.008), low relative lymphocyte count (p = 0.001), low relative eosinophil count (p = 0.006), high absolute and relative neutrophil count (both, p = 0.001) (Table 2), confirming the potential of these blood-derived parameters as biomarkers in immune checkpoint inhibition therapies [33]. Low neutrophil-to-lymphocyte ratio (NLR) and low derived NLR (dNLR) resulted also significantly associated with OS in patients treated with nivolumab (Table 2 and Additional file 2: Fig. S2), accordingly to our recently published data [34]. Focusing on CD8+ cells subsets, CD8+PD-1+ lymphocytes < 9.8% median frequency (p < 0.009) and CD8+PD-1+CD73+ lymphocytes < 2.3% median frequency (p < 0.001) were significantly associated with OS, whilst the proportion of CD8+T cells positive only to CD73 resulted no significantly associated with OS (Table 2). In a multivariate model, the median frequency of CD8+PD-1+CD73+ lymphocytes < 2.3% was also independently associated with longer overall survival [HR 2.17 (1.34–3.51); p = 0.002].

**Table 2:** Cox regression analysis

	HR (95% CI)
Sex	
Male vs female	0.92 (0.58–1.46) p = 0.73
Age	
> 62 vs < 62	0.70 (0.44–1.12) p = 0.13
BRAF	
mut vs wt	1.06 (0.66–1.70) p = 0.81
LDH	
High vs normal	1.62 (0.86–3.04) p = 0.13
Very high vs normal	4.64 (2.52–8.54) p < 0.0001
Brain metastasis	
Yes vs no	1.92 (1.18–3.11) p = 0.008
Previous lines of treatment	
2 vs 1	1.12 (0.62–2.02) p = 0.69
≥ 3 vs 1	1.38 (0.75–2.54) p = 0.30
Metastatic site	
Lung vs soft tissue	0.76 (0.30–1.87) p = 0.54
Visceral vs soft tissue	1.85 (0.93–3.65) p = 0.08
Absolute lymphocyte count	0.87 (0.60–1.25) p = 0.45
Relative lymphocyte count (≥ 20 vs < 20)	0.96 (0.93–0.98) p = 0.001
Relative monocyte count	0.94 (0.82–1.06) p = 0.32
Relative eosinophil count (≥ 1.5 vs < 1.5)	0.75 (0.62–0.92) p = 0.006
Absolute neutrophil count (≥ 8 vs < 8)	1.11 (1.04–1.17) p = 0.001
Relative neutrophil count (≥ 70 vs < 70)	1.04 (1.01–1.06) p = 0.001
NLR	1.04 (1.02–1.06) p < 0.0001
dNLR	1.09 (1.04–1.14) p < 0.0001
Tot CD8+ cells	
≥ 25.1 vs < 25.1	1.37 (0.86–2.18) p = 0.18
CD8+CD73+ cells (≥ 5.8 vs < 5.8)	1.55 (0.97–2.48) p = 0.07
CD8+PD-1+ cells (≥ 9.8 vs < 9.8)	1.86 (1.17–2.98) p = 0.009
CD8+PD-1+CD73+ cells (≥ 2.3 vs < 2.3)	2.25 (1.39–3.63) p = 0.001

*HR* hazard ratio, *CI* confidence interval, *LDH* lactate dehydrogenase, *NLR* neutrophil-to-lymphocyte ratio, *dNLR* derived NLR

**Fig. 2:**
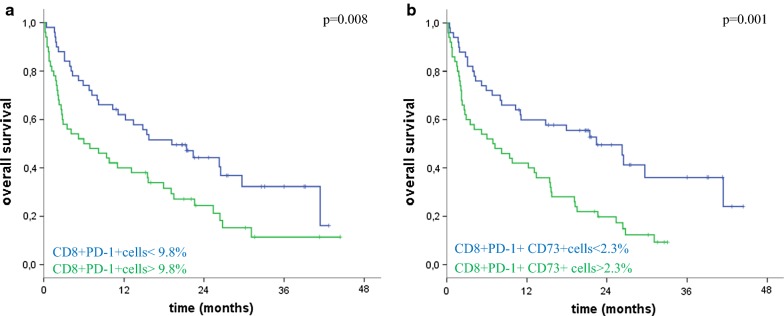
Kaplan–Meier curves showing the relation between the OS and the proportion of CD8 lymphocytes positive to PD-1 (**a**) together with CD73 (**b**) before treatment with nivolumab. P values were calculated by log-rank test

Median OS of patients, based on circulating CD8+ lymphocytes subsets at the cut-off value identified with the median, is reported in Table 3. Frequency of circulating CD8+T cells positive to PD-1 was inversely associated with survival (p = 0.008) (Table 3 and Fig. 2a). Notably, patients with low percentages of CD8+PD-1+CD73+ lymphocytes (< 2.3%) (n = 52/100) had an extended OS (22.4 months) compared to patients with higher CD8+PD-1+CD73+ lymphocytes frequency (6.9 months) (n = 48/100) (Table 3 and Fig. 2b). Frequency of CD8+PD-1+CD73+ lymphocytes resulted also significantly associated with progression-free survival (PFS) in these patients (Additional file 3: Table S1).

**Table 3:** OS according to the frequency of the reported cell populations

Cut-off (median frequency)	Median survival (months) (95% CI)	p value
CD8+ lymphocytes		
< 25.1%	15.4 (5.8–25.0)	0.18
> 25.1%	6.9 (0.1–13.7)	
CD8+ CD73 + lymphocytes		
< 5.8%	17.9 (4.9–30.9)	0.06
> 5.8%	8.0 (0.8–15.2)	
CD8+PD-1+ lymphocytes		
< 9.8%	19.2 (10.1–28.3)	0.008
> 9.8%	6.0 (0–12.8)	
CD8+PD-1+CD73+ lymphocytes		
< 2.3%	22.4 (13.6–31.2)	0.001
> 2.3%	6.9 (0.8–13.0)

### Association between baseline levels of circulating CD8+ CD73+ lymphocytes and clinical response to nivolumab

**Fig. 3:**
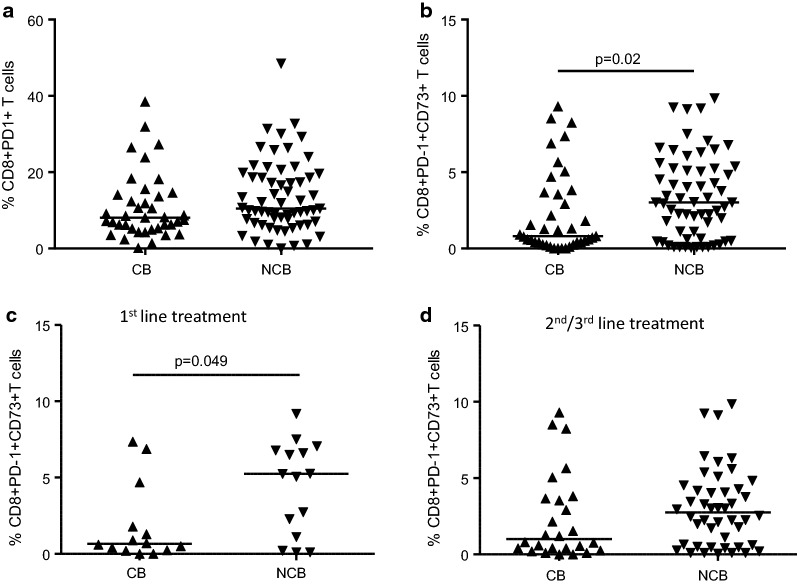
Baseline frequencies of CD8+PD-1+ lymphocytes (**a**) and CD8+PD-1+CD73+  lymphocytes (**b**) in patients groups based on clinical benefit to nivolumab treatment. Baseline frequencies of CD8+PD-1+CD73+ lymphocytes in 1st line treatment (**c**) and 2nd/3rd line treatment (**d**) patients subgroups based on clinical benefit to nivolumab therapy. *CB* clinical benefit, n = 39/100; *NCB* no-clinical benefit, n = 61/100. Statistical analysis was performed with Mann–Whitney test. Line indicates the median

Because our data indicate an association between the proportion of CD8+PD-1+CD73+ lymphocytes and survival, we investigated whether there was any correlation between these cells and clinical benefit to nivolumab. Patients were arbitrarily stratified in two groups based on clinical response to nivolumab therapy as detailed in “Materials and methods” section. No significant changes in CD8+PD-1+ lymphocytes frequencies were observed between patients with clinical benefit to nivolumab (CB group) and those without clinical benefit (NCB group) (p = 0.07; Fig. 3a). Instead, patients who experienced clinical benefit to nivolumab treatment (CB group) showed a lower pre-treatment median frequency of circulating CD8+PD-1+CD73+ lymphocytes (0.85%) than patients who progressed to nivolumab treatment (NCB group) (3.02%) (p = 0.02) (Fig. 3b).

Additionally, we also evaluated whether there was any correlation between these cells and clinical benefit in patients treated with 1st line nivolumab versus 2nd/3rd line nivolumab. The proportion of CD8+PD-1+CD73+ lymphocytes in patients treated with 1st line nivolumab (27/100 patients) resulted lower that those observed in patients treated with 2nd/3rd line nivolumab (73/100 patients) (1.8% median frequency vs 2.26% median frequency, respectively), although the difference was not statistically significant. Notably, among the patients treated with 1st line nivolumab we observed that the frequency of CD8+PD-1+CD73+ lymphocytes (0.65% median frequency) was significantly lower in patients with clinical benefit (13/27 patients) compared with those without clinical benefit (5.24% median frequency; 14/27 patients) (p = 0.049) (Fig. 3c). Among the group of patients treated with 2nd/3rd line nivolumab the frequency of CD8+PD-1+CD73+ lymphocytes in patients with clinical benefit (26/73 patients) was 1.0% vs 2.75% observed in patients without clinical benefit (47/63 patients) (Fig. 3d).

These results indicate that clinical response to nivolumab treatment is significantly associated with low pre-treatment CD8+PD-1+CD73+ lymphocytes levels and based on the number of previous treatments the frequency of these cells was significantly high in patients without clinical benefit treated with 1st line nivolumab.

## Discussion

In this study, we analysed the peripheral CD8+PD-1+ lymphocytes in melanoma patients before nivolumab treatment, exploring the proportion of cells positive to CD73. We found that baseline frequencies of CD8+PD-1+CD73+ lymphocytes in the peripheral blood of nivolumab treated-patients were associated with clinical benefit. In particular, patients with high baseline frequencies of circulating CD8+PD-1+CD73+ lymphocytes had poor outcome from nivolumab treatment. These results therefore suggest a potential role of peripheral CD8+ lymphocytes positive to CD73 in predicting anti-PD-1 therapeutic response in melanoma patients.

Anti-PD-1 agents, by preventing T cells inactivation in the tumor microenvironment, enhance anti-tumor response in cancer patients improving significantly overall survival. However, most patients do not respond to anti-PD-1 monoclonal antibodies, highlighting the need to understand the potential mechanisms of resistance to checkpoint therapy, useful to both improve the efficacy of the treatment and predict patients response. To date, some factors that correlate with clinical response to anti-PD-1 agents have been identified. A number of blood-derived factors including high relative eosinophil count and relative lymphocyte count, low dNLR, low LDH and absence of metastasis other than soft-tissue/lung have been associated with better OS in melanoma patients treated with anti-PD-1 agents [33, 34], as observed here. In addition, high mutational burden in human tumors, including melanoma, correlates with clinical response to PD-1 inhibitors [35]. However, although tumor mutational load, as well as PD-L1 expression on tumor cells, have proved to influence clinical response to PD-1 inhibitors, the heterogeneity of the tumor tissue and the dynamic nature of the signature associated with the tumor microenvironment, have prompted researchers to evaluate multiple factors that can contribute to provide a detailed characterization of patients immune system status. Correlations of different subsets of T cells with outcomes of cancer patients treated with immune checkpoint inhibitors have also been investigated. The presence of CD8+PD-1+ T cells at the tumor lesions (associated with tumor PD-L1 expression) [36–38] has been associated with therapeutic efficacy of anti-PD-1 agents. In non-small cell lung cancer (NSCLC) patients proliferation of peripheral blood CD8+T after PD-1-targeted therapy occur only in cells expressing PD-1, and this cells subpopulation is high in responding patients [39]. In melanoma patients expression of PD-1 on tumor-infiltrating CD8+T cells or on circulating T cells has been considered as a marker of tumor-reactive T cells [40–43]. More recently, it has been demonstrated that melanoma patients and NSCLC patients have a reduced percentage of circulating naïve T cells compared to controls [44]. To note, NSCLC patients with high central memory T cell (T_CM_) to effector T cell (T_Eff_) ratios, associated with inflamed tumors, showed longer progression-free survival to nivolumab [44]. Based on the associations of T cells states with response to immune checkpoint inhibitors, a detailed analysis of immune cells profile in melanoma tumors has been also reported [45]. In particular, responders lesions to checkpoint inhibitors are enriched of memory-like CD8+T cells whilst exhausted-like CD8+T cells are associated with no response [45]. Therefore, co-expression of multiple T cell disfunction markers can dramatically affect the effectiveness of checkpoint therapy. Here we examined the correlation of blood lymphocytes expressing CD73, together with PD-1, with OS and PFS in melanoma patients treated with nivolumab.

High expression of CD73 is associated with increased production, at the extracellular level, of the immunosuppressive mediator adenosine, that has been shown to exert suppressive effect on T cells [9, 10]. A large number of evidences indicate that inhibition of adenosine production or its activity improves the T cell-mediated anti-tumor responses, leading to tumor growth inhibition [10–13]. Elevated expression of CD73 on tumor cells and/or other tumor-infiltrating immune cells and/or other stroma cells has been associated with poor prognosis in cancer patients [23–27]. Furthermore, melanoma patients with increased tumor expression of CD73 show resistance to anti PD-1 agents or adoptive T cell therapy [46]. High tumor expression of CD73 has also been observed in BRAF-mutant melanoma patients [47]. CD73 has been detected also in serum of melanoma patients and high values of CD73 activity are inversely associated with response and OS in these patients undergoing nivolumab treatment [28]. Here, we expand our data determining the frequency of circulating CD8+ CD73+ lymphocytes at baseline in melanoma patients treated with nivolumab. We observed that high levels of CD8+ lymphocytes, expressing PD-1, positive to CD73 in the peripheral blood at baseline is associated with worse survival and poor clinical benefit to nivolumab. However, whether the associations of the abundance of circulating CD8 cells positive to CD73 and OS in melanoma patients might be prognostic or predictive for outcome after nivolumab treatment is not clear and needs further investigations.

An important limitation of this works is the lack of information on the functional involvement of CD73+ lymphocytes in the immune response against melanoma cells during nivolumab treatment. In the adenosine pathway, CD39, which produce the CD73-substrate AMP from ATP/ADP, together with TIM-3, identifies exhausted CD8+T cells with impaired production of TNF-α and IFN-γ [45], confirming that the co-expression of inhibitory molecules with PD-1 might limit the functions of these cells. CD73 on T cells has been associated with an exhausted phenotype in a mouse model of head and neck squamous cell carcinoma (HNSCC) and blockade of CD73 reverses the “exhausted” phenotype of T cells [48]. CD73, expressed by memory/naïve CD8+T cells within mouse tumors, by producing adenosine, may limit CD8+T‐cell differentiation to effector cells [49]. Here, we have only analysed associations between survival and frequency of CD73+/PD-1+ cells in the compartment of total CD3+CD8+T cells in the peripheral blood. Patients with clinical benefit to nivolumab showed lower proportion of CD8+PD-1+CD73+ T cells compared with patients with non-clinical benefit. Thus, the abundance of these cells seems to negatively impact the clinical outcomes in patients undergoing nivolumab treatment. This effect was particularly evident in first-line nivolumab patients, and to a lesser extent in patients treated with second/third line nivolumab, leading us to suppose that likely the expression of CD73 itself might be regulated on-treatment. An integrated analysis of CD73 expression together with other markers in CD8+ cell subpopulations, including CD8 T effector/memory cells, would provide a better understanding on the correlation of CD73+T cell status and clinical response to nivolumab as well as on the role of CD73 in controlling T cells behaviour. The high frequency of CD73-expressing CD8 T cells that we observed in patients non- responsive to nivolumab may indicate an alternative immunosuppressive mechanism induced by CD73-derived adenosine, which contribute to render these cells dysfunctional. As we have previously demonstrated [28], patients with high enzymatic activity of soluble CD73 showed low response rate to nivolumab. These data thus indicate that CD73, either in a soluble form and in a T-cell membrane-bound form, by producing adenosine could potentially counteract the clinical efficacy of PD-1 blockade, reinforcing the therapeutic potential of targeting adenosine signalling pathway to improve the efficacy of immunotherapeutic agents in cancer patients. In support, data in animal models, demonstrate that adenosine-targeted agents can enhance the efficacy of PD-1 and CTLA-4 blockade [29, 30], providing the basis of ongoing clinical trials testing new anti-CD73 agents and adenosine A2AR antagonist in combination with PD-1 or PD-L1 blockade [10, 13]. Although further experiments are needed to validate these results and clarify the role of these cells in regulating the response of T cells in patients undergoing immune checkpoint therapy, analysis of these cells in the peripheral blood of patients and among tumor-infiltrating lymphocytes (TILs) might provide additional insights in the status of T cells and prospectively it could integrate the value of other markers that influence the clinical response to immune therapeutics.

## Conclusions

In summary, we observed that frequency of CD8+PD-1+CD73+ T cells is significantly associated with OS of patients with metastatic melanoma. Low pre-treatment frequency of CD8+PD-1+CD73+ T cells in the peripheral blood of melanoma patients was associated with clinical benefit to nivolumab, indicating that, analysis of CD73 on circulating T cells at baseline may help to identify subsets of patients who most likely will benefit from nivolumab treatment. This study further reinforces the potential prognostic value of CD73 and encourages to explore anti-CD73 agents in combination with anti-PD-1 drugs to further improve immune response in melanoma patients.

## Supplementary information

**Additional file 1: Fig. S1.** Gating strategy to define CD8+ cell subpopulations in PBMC.

**Additional file 2: Fig. S2.** Kaplan–Meier OS curves of melanoma patient treated with nivolumab, according to the baseline derived neutrophils-to lymphocyte ratio (dNLR).

**Additional file 3: Table S1.** PFS according to the frequency of the reported cell populations.

## Data Availability

All data generated and/or analysed during this study are included in this published article and additional information is available upon request.

## Abbreviations

PD-1: Programmed cell death protein-1
CTLA-4: Cytotoxic T lymphocyte antigen-4
TIM3: T cell immunoglobulin and mucin domain-containing protein 3
LAG3: Lymphocyte activation gene 3 protein
TIGIT: T cell immune-receptor with immunoglobulin and ITIM domains
mAbs: Monoclonal antibodies
Tregs: T regulatory cells
MDCSs: myeloid-derived suppressor cells
DCs: Dendritic cells
ATP: Adenosine triphosphate
ADP: Adenosine-5′-diphosphate
AMP: Adenosine monophosphate
ADPR: ADP-ribose
NAD+: Nicotinamide adenine dinucleotide
NK: Natural killer
OS: Overall survival
PBMCs: Peripheral blood mononuclear cells
LDH: Lactate dehydrogenase
RECIST: Response evaluation criteria in solid tumors
CR: Complete response
PR: Partial response
SD: Stable disease
PD: Progressive disease
PE: Phycoerythrin
APC: Allophycocyanin
HR: Hazard ratio
AJCC: American Joint Committee on Cancer
NSCLC: Non-small cell lung cancer
HNSCC: Head and neck squamous cell carcinoma
TNF-α: Tumor necrosis factor-α
IFN-γ: Interferon-γ
TILs: Tumor-infiltrating lymphocytes
