# Supplementary figures and images for: Frequency of circulating CD8+CD73+T cells is associated with survival in nivolumab-treated melanoma patients
Source: J Transl Med. 2020 Mar 11;18:121. doi: 10.1186/s12967-020-02285-0 (PMC7065327; doi:10.1186/s12967-020-02285-0)

## Slide 1
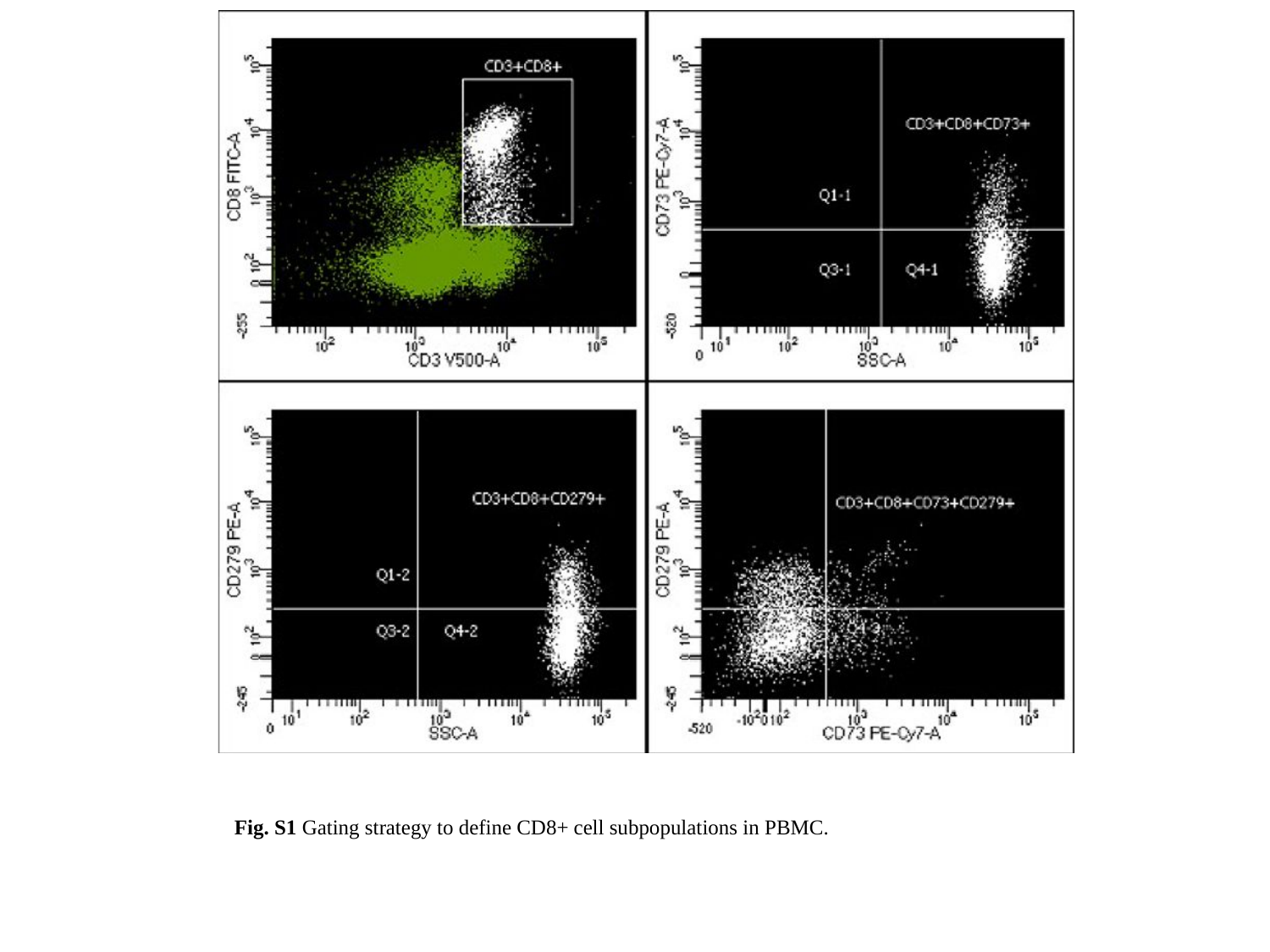

Fig. S1 Gating strategy to define CD8+ cell subpopulations in PBMC.

Supplement: Supplementary file 1 — Additional file 1: Fig. S1. Gating strategy to define CD8+ cell subpopulations in PBMC. [file 12967_2020_2285_MOESM1_ESM.pptx]
