# Supplementary material for: Frequency of circulating CD8+CD73+T cells is associated with survival in nivolumab-treated melanoma patients
Source: J Transl Med. 2020 Mar 11;18:121. doi: 10.1186/s12967-020-02285-0 (PMC7065327; doi:10.1186/s12967-020-02285-0)
Supplement: Supplementary file 2 — Additional file 2: Fig. S2. Kaplan–Meier OS curves of melanoma patient treated with nivolumab, according to the baseline derived neutrophils-to lymphocyte ratio (dNLR). [file 12967_2020_2285_MOESM2_ESM.pptx]

## Slide 1
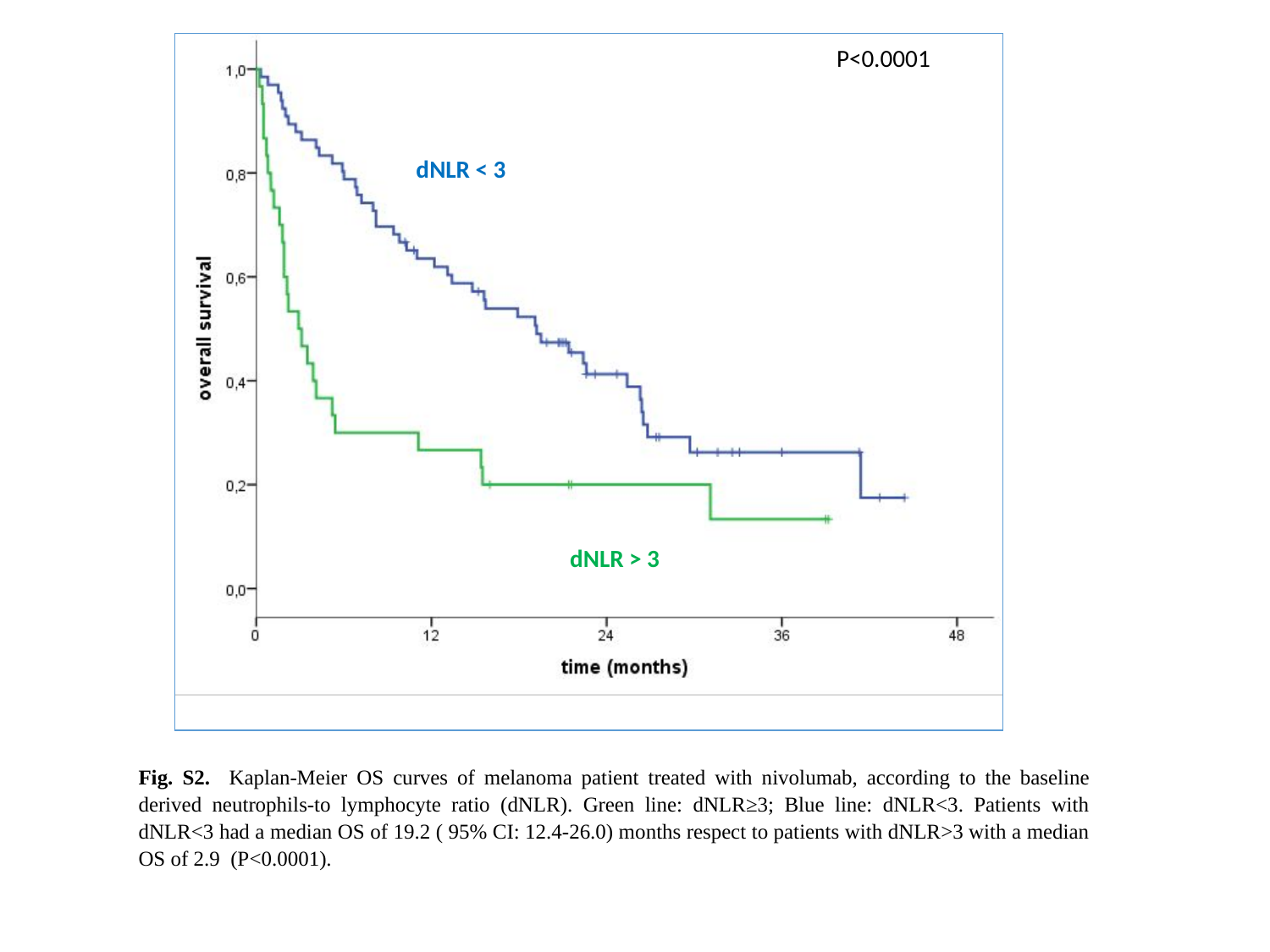

P<0.0001
dNLR < 3
dNLR > 3
Fig. S2. Kaplan-Meier OS curves of melanoma patient treated with nivolumab, according to the baseline derived neutrophils-to lymphocyte ratio (dNLR). Green line: dNLR≥3; Blue line: dNLR<3. Patients with dNLR<3 had a median OS of 19.2 ( 95% CI: 12.4-26.0) months respect to patients with dNLR>3 with a median OS of 2.9 (P<0.0001).
