# Supplementary material for: Frequency of circulating CD8+CD73+T cells is associated with survival in nivolumab-treated melanoma patients
Source: J Transl Med. 2020 Mar 11;18:121. doi: 10.1186/s12967-020-02285-0 (PMC7065327; doi:10.1186/s12967-020-02285-0)
Supplement: Supplementary file 3 — Additional file 3: Table S1. PFS according to the frequency of the reported cell populations. [file 12967_2020_2285_MOESM3_ESM.pdf]

Table S1 PFS according to the frequency of the reported cell populations

	**Cut-off (median frequency)**	**Median survival (months) (95%CI)**	**P value**
CD8+ lymphocytes	<25.1%<br>>25.1%	3.9 (0.8-7.0)<br>3.2 (1.1-5.3)	0.24
CD8+CD73+ lymphocytes	<5.8%<br>>5.8%	3.9 (0-10.1)<br>3.1 (0.9-5.3)	0.22
CD8+PD-1+ lymphocytes	<9.8%<br>>9.8%	5.9 (0.4-11.4)<br>2.6 (1.7-3.5)	0.02
CD8+PD-1+CD73+ lymphocytes	<2.3%<br>>2.3%	9.0 (0-19.9)<br>2.7 (2.1-3.3)	<0.0001
